# Self-buffering capacity of a human sulfatase for central nervous system delivery

**DOI:** 10.1038/s41598-021-86178-2

**Published:** 2021-03-24

**Authors:** Yi Wen, Nazila Salamat-Miller, Keethkumar Jain, Katherine Taylor

**Affiliations:** 1 Shire Pharmaceuticals (a Subsidiary of Takeda Pharmaceutical Company), 200 Shire Way, Lexington, MA 02421 USA; 2 Present Address: Lilly Research Laboratories, Eli Lilly and Company, Indianapolis, IN 46285 USA

**Keywords:** Enzymes, Proteins, Recombinant protein therapy, Drug delivery, Biologics, Protein delivery

## Abstract

Direct delivery of therapeutic enzymes to the Central Nervous System requires stringent formulation design. Not only should the formulation design consider the delicate balance of existing ions, proteins, and osmolality in the cerebrospinal fluid, it must also provide long term efficacy and stability for the enzyme. One fundamental approach to this predicament is designing formulations with no buffering species. In this study, we report a high concentration, saline-based formulation for a human sulfatase for its delivery into the intrathecal space. A high concentration formulation (≤ 40 mg/mL) was developed through a series of systematic studies that demonstrated the feasibility of a self-buffered formulation for this molecule. The self-buffering capacity phenomenon was found to be a product of both the protein itself and potentially the residual phosphates associated with the protein. To date, the self-buffered formulation for this molecule has been stable for up to 4 years when stored at 5 ± 3 °C, with no changes either in the pH values or other quality attributes of the molecule. The high concentration self-buffered protein formulation was also observed to be stable when exposed to multiple freeze–thaw cycles and was robust during in-use and agitation studies.

## Introduction

Enzyme replacement therapy targets rare genetic diseases that often exhibit various degrees of central nervous system (CNS) deterioration. Direct targeting of the CNS involves enzyme delivery to either intrathecal (IT), intracerebroventricular (ICV), or intracisternal space, where a delicate balance of ions, protein, and physiological pressure exists^1–3^. A thorough literature search for the formulations delivered to any of these spaces demonstrated that most of the molecules, specifically non-biologic anaesthetics, are formulated in sodium chloride and water solutions to avoid any disturbances in the cerebrospinal fluid^2^. Therefore, a suitable formulation design would need to be free of unnecessary species and adequately concentrated to ensure that limited volume of an appropriately formulated enzyme is introduced into the IT space.

In addition to the IT formulation design space requirements, another difficult task is to formulate an enzyme in a non-physiological environment while it maintains its long-term stability and efficacy; for biological macromolecules, due to the inherent unstable and complex nature, this is not a primitive task. As summarized by Warne, the formulation design space for antibodies commonly consists of histidine buffer with sucrose, in the pH range of 6.0 ± 0.4, with either polysorbate 20 or 80^4^. However, buffering species may not be unequivocally appropriate or required as proteins possess significant buffering capacity due to the presence of mainly numerous aspartic and glutamic acids as well as histidine residues. This inherent self-buffering capacity could be quite significant at high protein concentrations and has been reported to be sufficient to maintain long term and/or accelerated stability of several antibody drugs^5–8^. In addition to the significant buffering capacity from the amino acids, the inherent self-buffering capacity of each protein may also be partially due to the presence of residual buffering species that are somehow associated with each protein during expression and processing of the protein.

Considering the IT formulation design space constraints and the self-buffering phenomena, this study was initiated to formulate a sulfatase in a saline-based matrix. The study presented in this paper demonstrated that such approach to formulation is feasible, where the buffering capacity of the sulfatase was systematically studied and long-term stability and efficacy data were generated. Additionally, same approach was tested with a second protein in the sulfatase family, which additionally confirmed its self-buffering and long-term stability for CNS delivery. This paper is mainly focused on one enzyme (referred as target sulfatase) with reference to the second sulfatase, when appropriate. The target sulfatase is currently in a Phase 2b clinical program. The self-buffered formulation has been administrated to CNS using an intrathecal drug delivery^9,10^. Additionally, the wide distribution of the product in the brain parenchyma in animal models is reported^11,12^.

## Experimental section

### Material and methods

The proteins used in these studies were sulfatase enzymes, expressed and purified at Shire Pharmaceuticals (a subsidiary of Takeda Pharmaceutical Company, Lexington, MA). Protein solutions at pH 6.0 were obtained by ultrafiltration/diafiltration (UF/DF) processes; proteins were first UF/DF into either a pH 6.0 citrate–phosphate or phosphate buffer, followed by a second UF/DF into an iso-osmotic sodium chloride solution. The final concentrations of the sulfatase proteins were ≤ 40 mg/mL.

For polypeptide studies, poly-L-histidine HCl (molecular weight > 5000 Da) was obtained from Sigma (Saint Louis, MO). Poly-L-lysine HCl (x = 10, MW = 1600 Da), poly-L-arginine HCl (x = 30, MW = 5800 Da), poly-L-glutamic acid sodium salt (x = 20, MW = 3000 Da), and poly-L-aspartic acid sodium salt (x = 30, MW = 4100 Da) were purchased from Alamanda Polymers (Huntsville, AL). All polypeptides were used as received. Bovine serum albumin (BSA), Sodium Chloride (NaCl), monobasic sodium phosphate monohydrate (NaH_2_PO_4_.H_2_O), dibasic sodium phosphate heptahydrate (Na_2_HPO_4_.7H_2_O), citric acid monohydrate, 1 N hydrochloric acid (HCl), and 1 N sodium hydroxide (NaOH) were purchased from J.T. Baker (Phillipsburg, NJ). Double-deionized in-house water was used to prepare all buffer solutions.

### Determination of associated phosphate groups in each protein and preparation of equivalent phosphate buffer solutions

Based on literature and our internal data, it is known that the target and second sulfatases contain a fixed amount of divalent cation in their structure^13,14^. Additionally, we identified the presence of residual phosphates based on Inductively Coupled Plasma Mass Spectrometry (ICP-MS) data. While reported that each divalent cation is non-covalently coordinated to amino acid side chains in its vicinity^13,14^, we hypothesized that some residual phosphates (either originated from post-translational modification (expression) or the manufacturing processes) may at least be potentially associated with a divalent cation or be in some sort of equilibrium with the metal. We hypothesized this because our ICP-MS data demonstrated a higher number of phosphate molecules as compared to what was calculated to be expressed as the post-translational modification (it should be acknowledged that additional identified phosphate molecule may only or also be in coordination with positively charged amino acids). Therefore, an approximate relative molar ratio of phosphorus to a divalent cation was calculated to normalize their concentrations for each sulfatase concentration, type, and lot.

To determine the number and amount of divalent cation and phosphates associated with the sulfatase, several lots of the drug substance were subjected to a full elemental analysis by ICP-MS. Once the normalized concentrations of residual phosphates were determined, they were used to make “equivalent” phosphate buffer solutions (see Results).

### Determination of surface exposed residues in each protein

Based on the target formulation pH value of 6.0 and the pKa of amino acids close to this pH, three main amino acids of Asp, Glu, and His were selected for the analysis. The number of surface exposed Asp, Glu, and His was determined using crystal structures of both the target and second sulfatases. RasMol molecular graphics visualisation software (version 2.6) was used to determine the surface exposed residues^15^. For BSA (used as a model protein), the crystal structure was obtained from the Protein Data Bank (rcsb.org) using the PDB ID of 4F5S. The BSA crystal structure was uploaded into Molecular Operating Environment (version 2019.0101). The structure was prepared using the “QuickPrep” function and its properties were calculated using the “Property Calculation” function. If a residue had > 20% area exposed (res_exp), it was considered surface exposed. The residues with 10–30% area exposed were also inspected individually to confirm if their side chains are exposed.

### Preparation of equivalent phosphate buffers, sulfatases, stressed sulfatase samples, BSA, and polypeptide solutions

**Figure 1:**
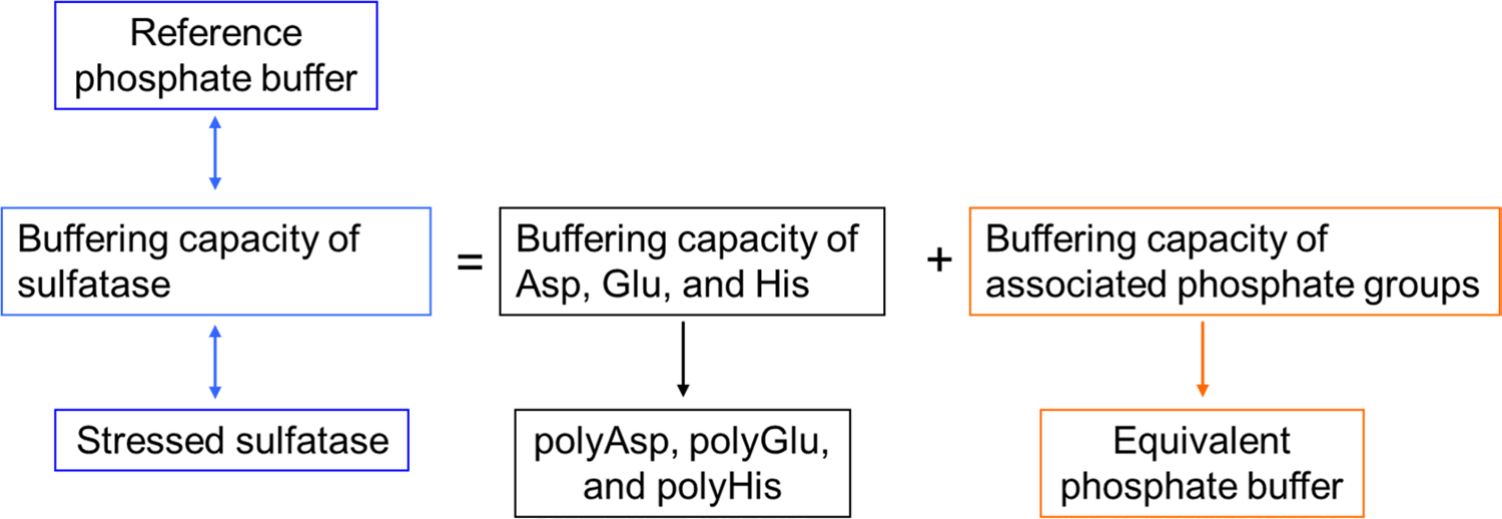
Schematic of study design.

To discern the potential factors contributing to the buffering capacity of the target sulfatase formulated in a sodium chloride solution, a study was designed (Fig. 1), where the protein’s buffering capacity was hypothesized to be a sum of the buffering capacities of protein’s amino acid residues (mainly Asp, Glu, and His due to the target formulation pH of 6.0) and any associated residual phosphate molecules. Additionally, in Fig. 1, the buffering capacity of the sulfatase was hypothetically cross-correlated to a reference phosphate buffer (i.e. what would be the buffering capacity of the sulfatase if measured in terms of a traditional phosphate buffer) and to structural changes of the sulfatase (i.e. what would happen to the buffering capacity if the structure of the sulfatase is altered).

To enable the study design and to make a reference for buffering capacity of our proteins, standard phosphate buffers were prepared (reference phosphate buffer). This study was followed by preparation of polypeptide solutions; similar to other studies in literature^5–7^ and as most amino acid residues do not possess ionizable side groups and are assumed not to contribute to the buffering capacity, only polypeptides including Asp, Glu, His, Arg, and Lys were selected. To simulate the amino acids’ buffering capacity (Fig. 1), corresponding polypeptides (polyAsp, polyGlu, polyHis, polyArg, and polyLys) were prepared (with the consideration that the pure polypeptide systems may not fully represent a complex protein matrix system). The preparation studies were concluded by making equivalent phosphate buffer solutions, the concentrations of which were calculated both using ICP-MS data and the associated phosphates described earlier in the manuscript (referred as equivalent phosphate buffer). Additionally, a degraded solution of target sulfatase was prepared (heat and pH-induced) to be compared to the non-stress molecule in terms of buffering capacity (Fig. 1).

Following the solutions preparation and to systematically investigate the buffering capacity of target sulfatase as depicted in Fig. 1, a series of titration studies were performed: (1) the titration studies of both reference and equivalent phosphate buffer solutions, (2) the titration studies of the target and stressed sulfatase at different protein concentrations, and (3) the titration studies of the polypeptide solutions. In addition, as a fourth titration experiment, the buffering capacities of a second sulfatase and BSA were compared with that of the target sulfatase to further explore the relationship between the amino acid composition (i.e. His, Asp, and Glu) and buffering capacity.

#### Titration studies of reference and equivalent phosphate buffer solutions

Phosphate buffer solutions at 50, 25, 10, 5, 2.1, 1.6, 1.05, 0.53, and 0.26 mM were prepared using monobasic and dibasic sodium phosphate salts. The pH of each solution was adjusted to a target value of 6.0 with either HCl or NaOH, if needed. The solutions were prepared to identify the buffering capacity of both reference phosphate buffers (at 50, 25, 10, and 5 mM) and equivalent phosphate buffers (at 1.05, 0.79, 0.52, 0.26, and 0.13 mM, Table S1). The equivalent phosphate buffer concentrations were derived from normalized protein and phosphorous to divalent cation ratio calculations.

#### Titration studies of target and stressed target sulfatase at different protein concentrations

The drug substance, at ≤ 40 mg/mL, was diluted with saline to obtain protein solutions at 38, 30, 20, 10, and 5 mg/mL. To prepare the heat and pH stressed sulfatase samples, the drug substance was dialyzed into a pH 7.0, iso-osmotic phosphate buffer. The samples were stored at 50 °C for 2 weeks (10 mL in a 20 mL Type I glass vial); this condition was previously shown to cause aggregation of the target sulfatase and the associated loss of activity (unpublished internal data). After the heat treatment, the stressed samples were dialyzed into a citrate–phosphate buffer at pH 6.0 with 137 mM sodium chloride, followed by a second dialysis into an iso-osmotic sodium chloride solution (Slide-A-Lyzer G2 dialysis cassette, 3,500 Da MWCO, Pierce Biotechnology) with three buffer exchanges. The stressed sample was diluted in iso-osmotic sodium chloride solution to 40, 30, 20, 10, and 5 mg/mL for the titration studies.

#### Titration studies of the polypeptide solutions

To prepare the polypeptide solution, a stock solution of 8 mg/mL of different polypeptides (polyHis, polyLys, polyArg, polyGlu, polyAsp) in an iso-osmotic sodium chloride solution was individually prepared. The pH of each solution was adjusted to 6.0 using either 1 N HCl or 1 N NaOH. Polypeptide solutions at approximately 4.0, 2.0, 1.0, and 0.5 mg/mL were prepared by diluting the stock solution before titration. These polypeptide concentrations were selected to cover the calculated molar concentrations of Asp, Glu, His, Arg, and Lys residues in the protein solutions that were studied.

#### Titration studies of the second sulfatase and BSA

To generate the samples for titration studies of the second sulfatase, a 20 mg/mL sample was concentrated to ≤ 40 mg/mL by centrifugation (4000*g*, 15 min) using a centrifugal filter unit (Amicon Ultra, 10 K MWCO, Millipore). The protein solutions at 36, 30, 20, 10, and 5 mg/mL were prepared by diluting the dialyzed protein with iso-osmotic sodium chloride solution.

The BSA solutions were prepared as a model system to which the buffering capacity of the two sulfatases were compared. To prepare the BSA solutions in iso-osmotic sodium chloride solution at pH 6.0 for the titration studies, BSA was dissolved in MilliQ water at 40 mg/mL. The BSA solution was then treated similarly to the UF/DF process of the target sulfatase; it was first dialyzed into a phosphate-citrate buffer at pH 6.0 with 137 mM NaCl (Slide-A-Lyzer G2 dialysis cassette) followed by the second dialysis into an iso-osmotic sodium chloride solution (Slide-A-Lyzer G2 dialysis cassette) with three buffer exchanges. The obtained BSA solution was diluted with saline to prepare solutions at 40, 30, 20, 10, and 5 mg/mL for titration studies.

### Titration of reference and equivalent phosphate buffers, proteins, and polypeptides solutions

For acid titration studies, 10 mL of each solution (Materials) was transferred into a 20 mL beaker. During pH measurements, the solution was constantly stirred using a magnetic stirrer to facilitate equilibrium. For acid titration studies, 1 N HCl was added to the 10 mL solution at increments of 1 µL or more (with a maximum volume of ~ 10 µL) until the target pH value was achieved for solutions at each concentration. After the addition of the HCl titrant, the solution was allowed to equilibrate before pH value was recorded (ORION3 STAR pH meter, Thermo Scientific). The titration process was ended when 1-unit pH change was achieved (for example from pH 6.0 to pH 5.0).

For base titration studies, 1 N NaOH was added to the previously acid titrated sulfatase solution to bring the pH back to 6.0. Since minimal volume was introduced (a total of 5–50 µL per 10 mL), the concentration and volume were not further adjusted to calculate the buffering capacity during base titration. After the pH was stabilized, 1 N NaOH was added at the increments of 1 µL or more (with a maximum volume of ~ 10 µL) until 1-unit pH change was achieved for solutions at each concentration (for example from pH 6.0 to pH 7.0). The pH value was recorded after each increment addition of NaOH. For standard phosphate buffers and polypeptide solutions, a new solution was used for the base titration studies.

The titration curve was constructed by plotting the measured pH values versus the amount (µmole) of acid or base added for each solution. Buffering capacity was calculated using the following equation within approximately linear range of the titration curve^8^:

$$\beta =\frac{M}{\Delta \mathrm{pH}*\mathrm{V}}$$

where β is buffering capacity, M is amount of titrant used (µmole), ∆pH is pH change (~ 1 unit), and V is volume of protein solution that was titrated (10 mL).

### Sulfatase long-term stability studies

Studies were conducted to evaluate the stability of self -buffered sulfatase drug substance and drug product formulations at ≤ 40 mg/mL. The stability samples were tested with assays that monitored purity, potency, content, pH, and other general quality attributes per the ICH guidelines. For the drug substance stability studies, the stability samples were packaged into polycarbonate vials. The stability was evaluated at the long-term storage condition of ≤ − 65 °C over 48 months. In addition, accelerated (5 ± 3 °C) and stress conditions (25 ± 2 °C) were also evaluated over 6 months and 3 months, respectively. For the drug product stability studies, the samples were filled into Type I borosilicate glass vials with fluororesin-coated serum rubber stoppers and aluminium overseals. The stability was evaluated at the long-term storage condition of 5 ± 3 °C over 48 months. In addition, accelerated (25 ± 2 °C) and stress conditions (40 ± 2 °C) were also evaluated over 6 months and 3 months, respectively.

### Sulfatase freeze–thaw studies

Studies were conducted to evaluate the effect of multiple freeze–thaw cycles on the stability of the sulfatase. Small scale freeze–thaw studies included three cycles of freezing to − 50 °C and thawing to 25 °C at a fast freeze rate and either a fast or slow thaw rate. At-scale freeze–thaw studies were also performed by freezing at ≤ − 65 °C for a minimum of 48 h followed by thawing at either ambient temperature conditions, 5 ± 3 °C, or water bath set at 37 °C. The quality of frozen and thawed sulfatase was assessed for purity, potency, content, and other quality attributes.

### In-use stability studies

Several studies were conducted to assess the in-use stability of sulfatase drug product as prepared for clinical use through an intrathecal drug delivery device. Individual studies were conducted to evaluate the compatibility and stability of the target sulfatase during in-use. The quality of in-use samples was assessed for purity, potency, content, and general quality attributes such as pH values and appearance.

### Agitation studies

Shaking stress studies were performed to examine the susceptibility of the target sulfatase to agitation-induced aggregate/particulate formation using a stress model in the absence of any buffering species. The target sulfatase drug product vials were placed in an orbital shaker in either upright or horizontal orientation, secured inside a secondary container, and agitated at 220 rpm for 48 h. The quality of agitated samples was assessed for purity, potency, content, and other quality attributes such as pH values and appearance.

## Results

### Determination of associated phosphate groups in each sulfatase

Full elemental analysis of multiple lots of the target sulfatase, by ICP-MS, demonstrated a 29 ppm of phosphorus and 13 ppm of a divalent cation on average (Table S2). Therefore, the molar ratio of phosphorus to the cation was calculated to be ~ 2.9. Since each target sulfatase dimer has one cation molecule, the number of phosphate groups associated with the sulfatase monomer of the cation was approximated to be 1.5. Therefore, the equivalent phosphate buffer solutions were prepared (Materials) at 1.5 times of protein molar concentration (Table S1).

### Titration studies of reference and equivalent phosphate buffer solutions

Theoretically, the buffer capacity is obtained by using a tangent at a given pH from a titration curve. In practice, the titration curve can be fitted by a straight line over a short pH range. The slope of the straight line can be used as the buffering capacity for that pH range. In this study, the buffering capacity was defined as the amount of acid or base needed to change the pH of a buffer solution by one pH unit. Additionally, buffering capacity was calculated using the required micromolar amount of acid (HCl) or base (NaOH) for that pH unit change per 1 mL of the protein solution (μmole/pH-mL). According to this definition, buffering capacity is linearly related to the buffer concentration.

**Figure 2:**
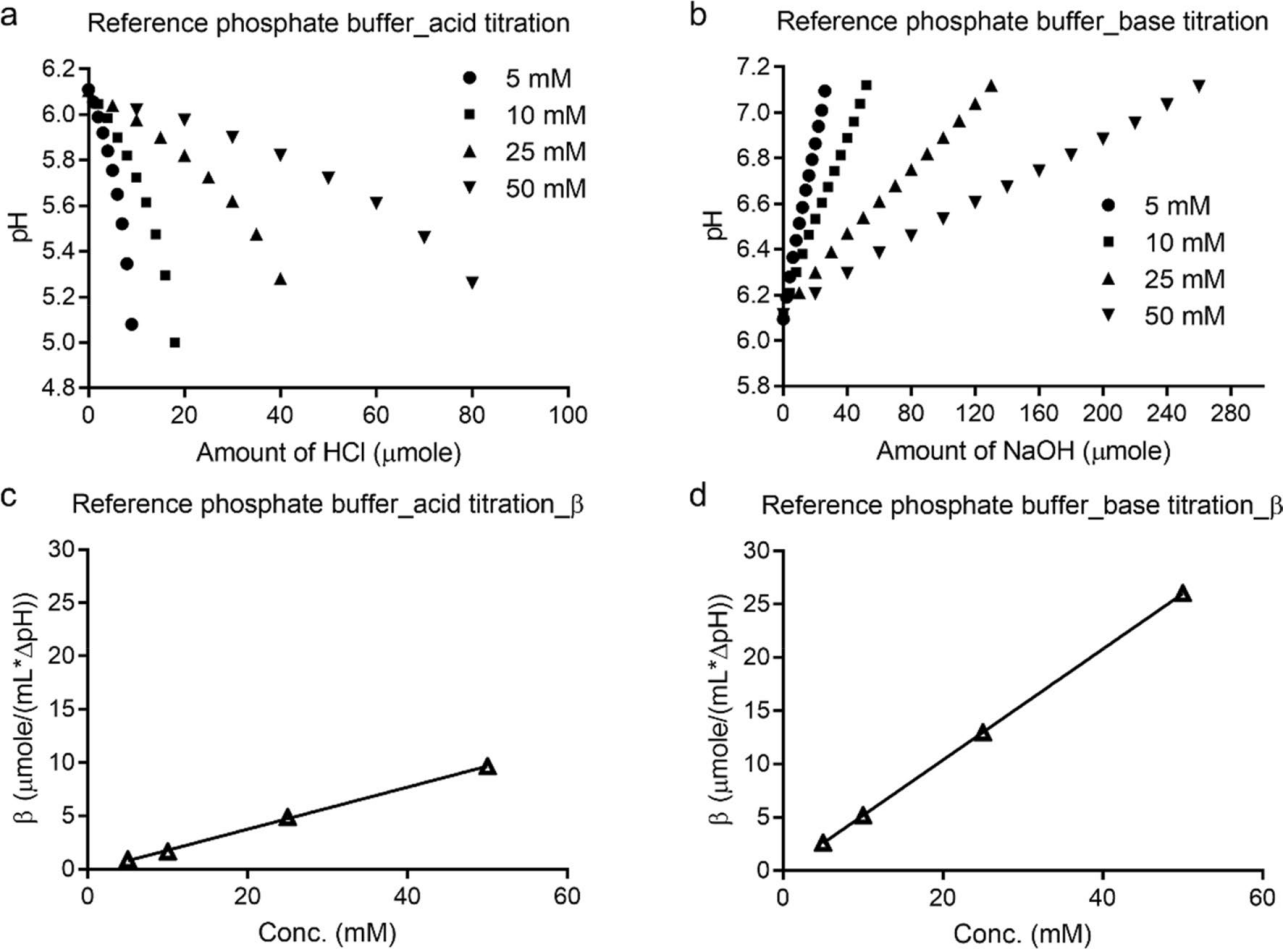
Acid or base titration curves and buffering capacity of reference phosphate buffers at different concentrations.

The phosphate buffers, prepared in the study, possessed strong buffering capacities during acid or base titration in a concentration dependent manner (Fig. 2, Figure S1). The buffering capacity towards the base titration was observed to be higher than that towards acid titration. This is not unexpected because a buffer solution demonstrates its highest buffering capacity over the 1-unit change around its pKa (pKa ± 1). The pKa of monobasic sodium phosphate is 7.21. Therefore, the buffering capacity calculated during base titration (from pH 6.0 to 7.0, which was in closer vicinity to the pKa), was higher than the buffering capacity during acid titration (the acid titration from pH 6.0 to 5.0).

### Titration studies of target and stressed target sulfatase at different protein concentrations

**Figure 3:**
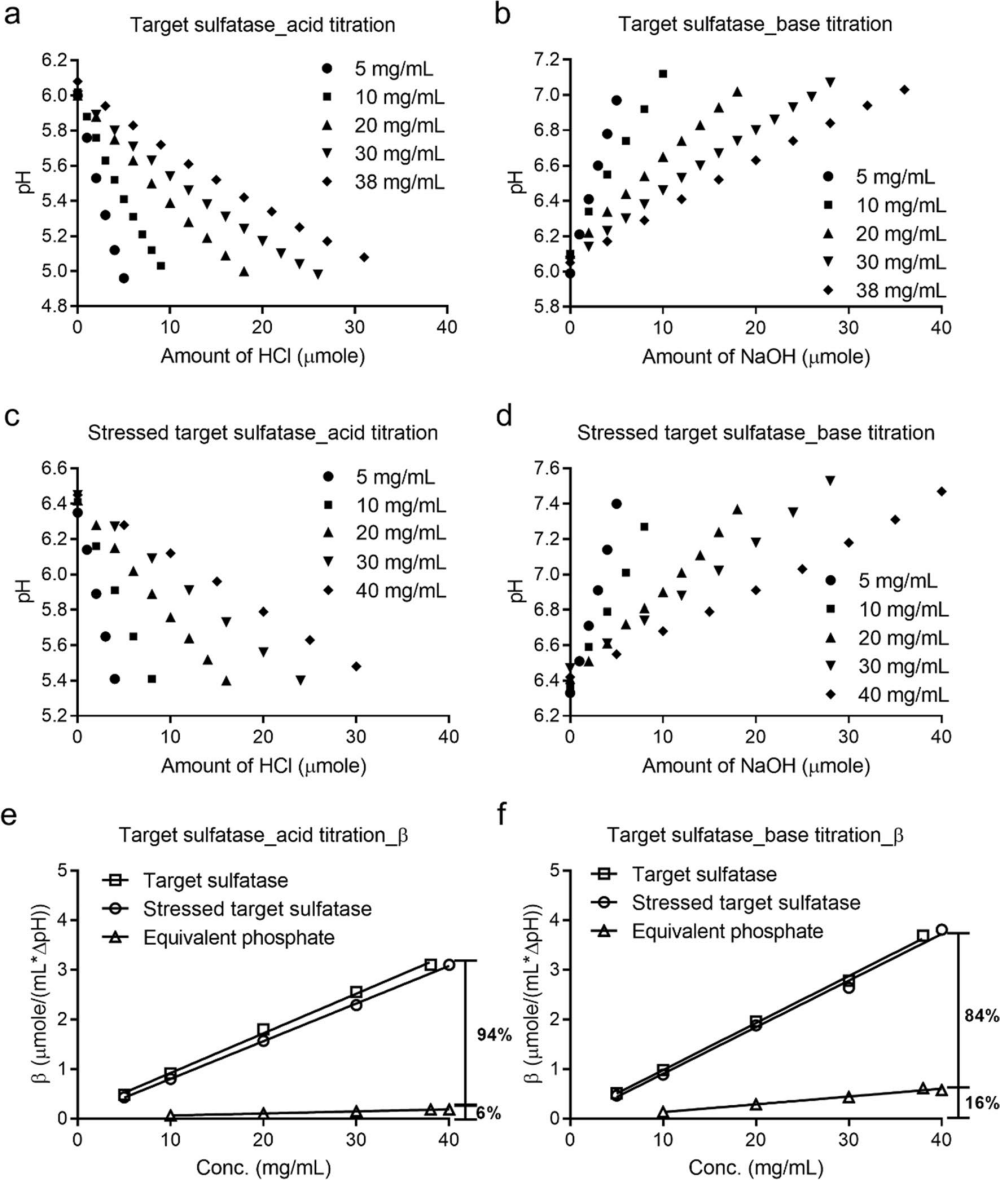
Acid or base titration curves and buffering capacity of target and stressed target sulfatases at different concentrations.

The buffering capacity of the target sulfatase was concentration dependent. At 30 mg/mL, the buffering capacity of the sulfatase was 2.6 μmole/pH-mL or 2.8 μmole/pH-mL when titrated by acid or base, respectively (Fig. 3). This meant that in order to achieve the same level of the protein buffering capacity in the phosphate buffers, approximately 13.9 mM phosphate buffer during acid titration or ~ 5.4 mM phosphate buffer during base titration, respectively, are required (Fig. 2). Hence, the target sulfatase at 30 mg/mL possessed a buffering capacity equivalent to either a 13.9 mM or 5.4 mM reference phosphate buffer during the acid or base titration at pH 6.0, respectively. However, since ~ 1.5 phosphate groups were hypothesized to be either associated with each sulfatase molecule or the divalent cation within, it was reasonable to speculate that the buffering capacity of the sulfatase came from both amino acid residues and these residual associated phosphate groups.

At 30 mg/mL, the molar concentration of the associated phosphate was ~ 0.8 mM (Table S1), which provided 0.15 or 0.44 μmole/pH-mL for either acid or base titration respectively. Therefore, the associated phosphates contributed to approximately 6% (0.15/2.6) or 16% (0.44/2.8) buffering capacity during acid titration or base titration, respectively (Fig. 3e,f).

In a similar manner, the stressed sulfatase, at 30 mg/mL, was titrated to investigate whether there would be any difference in the buffering capacity that may be related to the physical aggregation and/or conformational aspects of the sulfatase. The data demonstrated a comparable buffering capacity of 2.3 or 2.6 μmole/pH-mL during acid or base titration, respectively (Fig. 3). This suggested that the total polar surface-exposed amino acid composition may not have changed post stress even though the previous knowledge on the stressed molecule demonstrated aggregation, fragmentation, and loss of specific activity post such stress (data not shown). It should be noted that the limited analysis of the stressed molecule by near UV Circular Dichroism (CD) suggested some type of fingerprint tertiary conformational change (Figure S2). However, the observed changes did not translate to loss of buffering capacity; the buffering capacity data demonstrated no apparent change post the thermal stress.

### Titration studies of the polypeptide solutions

**Figure 4:**
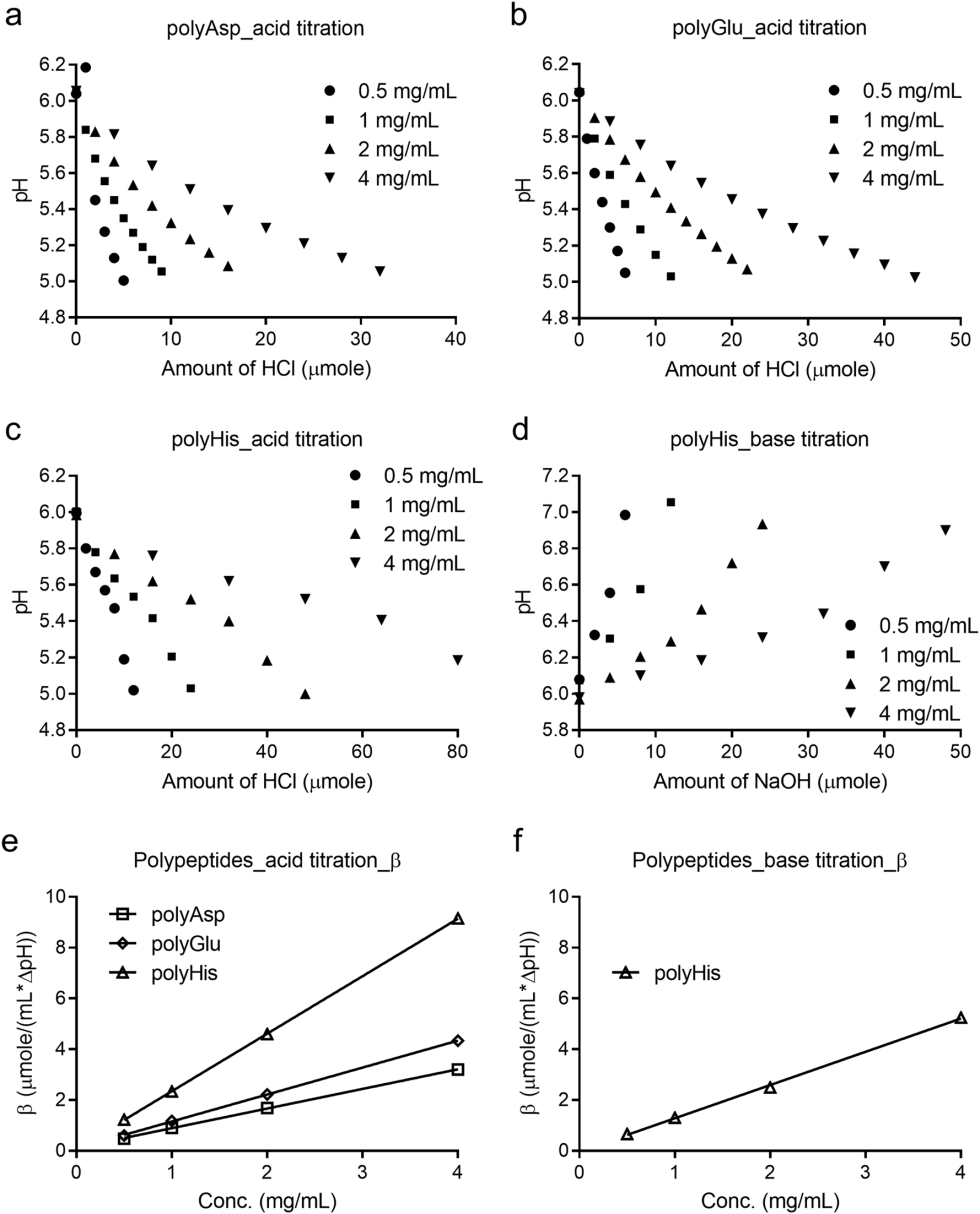
Acid or base titration curves and buffering capacity of polypeptide solutions at different polypeptide concentrations.

The titration studies of polypeptide solutions showed that each kind of amino acid had different buffering capacity during acid and base titrations. PolyAsp and polyGlu showed significant buffering capacities during acid titration (Fig. 4), but negligible buffering capacities with base titration (Figure S3). PolyArg and polyLys showed no buffering capacities during both acid and base titration (Figure S3). In addition, PolyHis showed noticeable buffering capacity during both acid titration and base titration (Fig. 4). The differences in buffering capacity values are most likely originated from the polypeptides’ pKa’s, where a higher buffering capacity was observed when the polypeptide was titrated toward a pH that was closer to its pKa. When the polypeptide was titrated towards a pH value that was away from the pKa, the polypeptide’s buffering capacity became negligible. For example, polyHis had the strongest buffering capacity during both acid and base titrations due to the overlap between the studied titration pH range (5.0 to 7.0) and its optimal buffering zone (pKa ± 1, pKa ~ 6.5)^16,17^. Although the exact pKa’s of the residues in each polypeptide remain to be determined, it may be reasonable to speculate that the polar surface exposed amino acids may possess a pKa close to their corresponding amino acid residues in proteins^17^. Any deviation from this proximity is then may be, at least partially, attributed to the tertiary structure of the protein (as the polypeptide do not possess any tertiary structure).

### Titration studies of the second sulfatase and BSA

**Figure 5:**
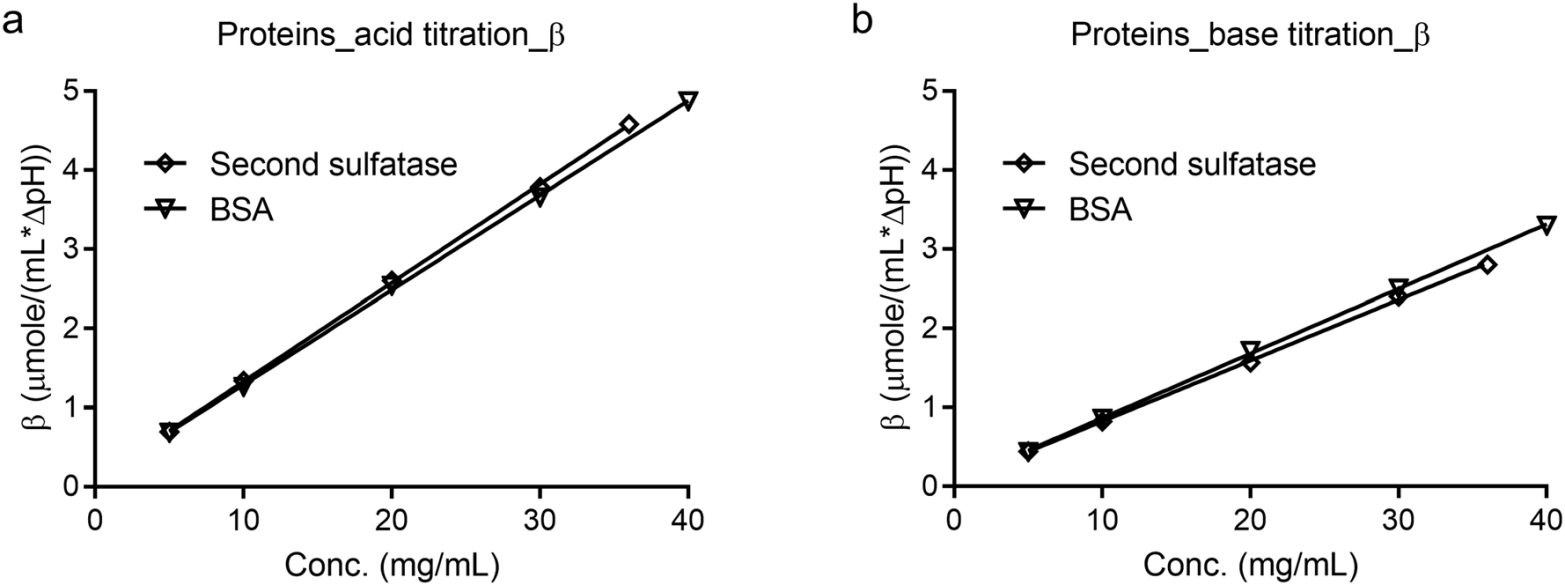
Acid or base summary of the buffering capacity of the second sulfatase and BSA at different protein concentrations.

The titration results of the second sulfatase and BSA demonstrated that their buffering capacity was also linearly related to their concentrations (Fig. 5, S4, and S5).

### Sulfatase stability studies

**Figure 6:**
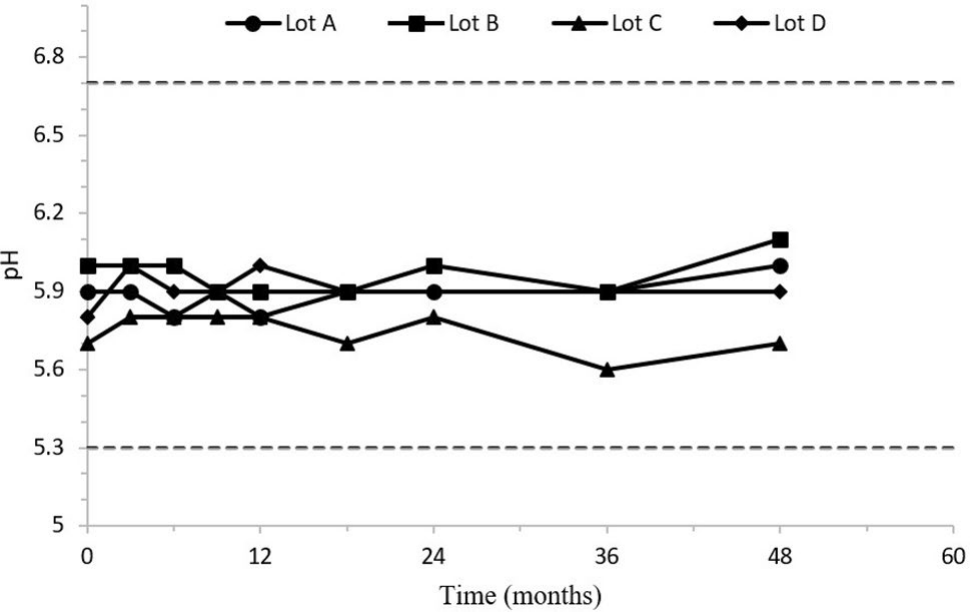
pH Values of four drug product lots of the target sulfatase as a function of time when stored for 48 months at long term storage condition of 5 ± 3 °C.

The stability data available to date demonstrate that the high concentration self-buffered sulfatase formulation continues to meet the target criteria for the quality attributes tested for both drug substance and drug product after 48 months at long-term storage conditions. The pH of the sulfatase formulation was maintained throughout the stability studies (Fig. 6). The data in Table 1 are presented as example data for one lot of the drug product. No changes in the purity of sulfatase has been observed by chromatographic and electrophoretic methods of SE-HPLC, SDS-PAGE, and peptide mapping over time when stored at the intended long-term storage condition. The structural integrity of the glycans, the potency, appearance, and content of sulfatase has also been maintained.

**Table 1:** Long-term stability data for one representative lot of the target sulfatase drug product.

Test	Month								
	Baseline	3	6	9	12	18	24	36	48
Size exclusion HPLC (% main peak area)	> 98%	> 98%	> 98%	> 98%	> 98%	> 98%	> 98%	> 98%	> 98%
RP-HPLC (% main peak area)	> 99%	> 99%	> 99%	> 99%	> 99%	> 99%	> 99%	> 99%	> 99%
SDS-PAGE (coomassie:reduced)	Conform	Conform	Conform	Conform	Conform	Conform	Conform	Conform	Conform
Specific activity (U/mg)	> 50	> 50	> 50	> 50	> 50	> 50	> 50	> 50	> 50
pH	5.8	6.0	5.9	5.9	6.0	5.9	5.9	5.9	5.9

It should be noted that as a part of the stability program, the stability of the target sulfatase was also tested at different temperatures. At the accelerated storage conditions (25 ± 2 °C), no significant changes to any quality attribute was observed following 6 months of storage. At the stress storage conditions (40 ± 2 °C), few changes were observed in the SE-HPLC, SDS-PAGE, specific activity, and the peptide map profiles but no change in the pH values was observed. No changes in any other quality attributes were observed after 3 months storage at the stress storage conditions. The changes discussed were not a function of pH change but due to the exposure of the molecule to a high temperature.

### Sulfatase freeze–thaw studies

The stability of the self-buffered target sulfatase was maintained after freezing to ≤ − 65 °C and thawing at either controlled room temperature, 5 ± 3 °C, or in a water bath suggesting that thawing temperature had no impact on the quality of the thawed product. It was also observed that the sulfatase was not susceptible to freeze–thaw induced degradation when frozen and thawed at different controlled rates. No change in the percent main peak area (> 98% by SEC) or pH was observed after freeze–thaw cycles (data not shown).

### In-use stability studies

The robustness of the formulation solution for the target sulfatase was further demonstrated by the in-use study. The sulfatase drug product was held in the CNS-dedicated administration device for up to 60 min with no impact to product quality. Additionally, the sulfatase was held in the delivery syringe for up to 8 h with no impact to product quality. No change in the pH, or any other quality attributes of the product was observed at any point that further supported the robustness of the self-buffered formulation during dose administration. The data for the pH values are summarized in Table 2.

**Table 2:** pH robustness data of the target sulfatase during in-use and agitation studies.

Study description	pH	
**Sulfatase drug product held in the CNS-dedicated device**	**Baseline**	**60 min**
	6.0	6.0
**Sulfatase drug product held in the delivery syringe for 8 h**	**Baseline**	**8 h**
	6.0	6.0
**Sulfatase drug product subjected to agitation stress**	**Baseline**	**48 h**
Upright orientation	5.9	5.8
Horizontal orientation	5.9	5.9

### Agitation studies

Shaking stress had no impact on any critical quality attributes of the target sulfatase over a period of 48 h in either vial orientation. The pH of the sulfatase formulation was maintained throughout the agitation (Table 2). It should be noted that the final product formulation solution contained minor amount of a surfactant to alleviate any adsorption- or aggregation-related protein loss.

## Discussion

In this study, the self-buffering capacity of two sulfatases are reported, quantified, and compared to a model protein BSA. In all studies, the buffering capacity was linearly related to the protein concentration (R^2^ > 0.995). The results demonstrated that self-buffering capacity may be a universal property for proteins, including monoclonal antibodies and enzymes^5–7,18^. More importantly, the self-buffered protein formulation exhibited exceptional stability during long term and accelerated storage conditions, it was also stable post multiple freeze–thaw cycles, and it was robust during in-use and agitation stability studies (Fig. 6, Tables 1, 2).

Such stability was also reported in previous publications on mAbs. Gokarn et al*.* reported self-buffered formulation that showed comparable long term and accelerated stability to conventionally-buffered (acetate and glutamate) formulations^5^. Additionally, Bahrenburg et al*.* demonstrated high temperature and shaking stress stability of self-buffered-formulation^6^, and Garidel et al*.* described accelerated high temperature and freeze-drying stability of such formulations^7^. The studies also pointed out that the buffering capacity of proteins may be mainly attributed to Asp, Glu, and His residues, which was observed in our studies (Fig. 4, Figure S3).

Although, it has been reported that monoclonal antibodies have comparable buffering capacities due to their similar molecular weight and composition, we have not lost sight of the reported buffering capacity variation in this field; for instance, both Gokarn et al. and Bahrenburg et al*.* reported approximately 25% variation of buffering capacity in the pH region of 5.0–6.0 for a total of 9 antibodies studied^5,6^. In addition to the molecular composition and conformation, variation in buffering capacity might stem from residual buffering species originated from the process, such as acetate and glutamate^5^. The self-buffering capacity may not likely be the case for proteins of different molecular weight, composition, and conformation. Indeed, Lamanda et al. described dramatically different buffering capacities of amyloglucosidase, lysozyme, and α-amylase solutions in water^18^. The optimal buffering of amyloglucosidase and α-amylase were found to be at pH 4.4 and pH 6.3 respectively. In contrast, lysozome did not show buffering capacity even at a concentration of 340 μM. In addition, the buffering capacity of purified salivary proteins, collected at different times of a day, varied, which was attributed to the different protein composition throughout of the day^18^. In our study, even within the same sulfatase family, we observed buffering capacity values of 2.4 and 5.0 μmole/pH-mL for the target and second sulfatase at 0.5 mM, respectively, during acid titration (Table S3). Therefore, a larger set of protein molecules and any potential residual buffering species need to be studied to further understand the self-buffering capacity phenomenon. Considering the potential structural and experimental variations, it should also be noted that the residual coordinated conventional buffering species, such as acetate and phosphates, introduced during upstream and downstream manufacturing process, also contribute to the observed buffering capacity. The latter is shown in the current study; the residual phosphates were either present on the molecular structure of the proteins or potentially coordinated with the inherent divalent cations present in the two studied sulfatases.

We sought to explore the correlation between amino acid composition and buffering capacity using the target and second sulfatases in this study (Table S3). In this study, we sought to evaluate our initial hypothesis that the protein’s total buffering capacity is assumed to be a sum of mostly the buffering capacities of protein’s amino acid residues (Asp, Glu, and His) and any associated residual phosphate molecules (Fig. 1).

The surface-exposure of amino acids were considered as the target and second sulfatases had a total of 63 and 79 Asp, Glu, and His residues, respectively. However, more than likely, the amino acid residues that were not solvent exposed would not have contributed to the observed buffering capacity. Hence, using only the surface-exposed amino acid residues was deemed more relevant to the calculations. To this point, the target and second sulfatases had a total of 36 and 59 surface exposed Asp, Glu, and His amino acids, respectively, as obtained from their corresponding crystal structures. As shown in Table S3, the calculated ratios of the second to target sulfatase with respect to their buffering capacities, number of total amino acids, and number of surface-exposed amino acids during acid titration were 2.2, 1.3, and 1.6, respectively. The ratios for similar parameters during base titration were 1.2, 0.8, and 1.3, respectively. Therefore, using only the solvent exposed amino acid residues improved the correlation between the buffering capacity and the number of amino acids. However, if indeed the buffering capacity was only a function of amino acid composition alone, the ratio of buffering capacity values should have been equal to the ratio of number of contributing amino acid residues. At a protein concentration of 0.5 mM (~ 30 mg/mL), the target and second sulfatases possessed a total buffering capacity values of 2.4 and 5.0 μmole/pH-mL, respectively, when titrated with an acid. Since theoretically ~ 6% of the buffering capacity for target sulfatase was attributed to the 1.5 associated residual phosphate molecules, the buffering capacity from total amino acid residues should have been 2.2 μmole/pH-mL for the target sulfatase. The 2.2 μmole/pH-mL is closer to the experimentally-determined value. This conclusion supported our initial hypothesis (Fig. 1) and that the total experimental buffering capacity of the target sulfatase was observed to be fairly close to the sum of the buffering capacities of corresponding surface exposed amino acid residues (Asp, Glu, and His from polypeptides) and the associated phosphates (estimated from equivalent phosphate buffer) (Fig. 1 and Table S3). The experimentally determined total buffering capacity of the target sulfatase was 5.1 μmole/pH-mL and the sum of the calculated buffering capacities was determined to be 5.6 μmole/pH-mL (Table 3). A similar relationship was also observed for the second sulfatase and BSA (to a lesser extent for BSA). The experimentally determined total buffering capacities for the target and second sulfatases were within 10% of their calculated values (Table 3). In this study, a good correlation was observed between the experimental and calculated buffering capacity values. This approach in general may help in providing an estimate of the buffering capacity of a given protein; however, as discussed, a larger set of molecules needs to be studied to further confirm the hypothesis.

**Table 3:** Comparison of the experimental and calculated buffering capacities.

Protein	Buffering capacity of the associated phosphates (estimated from equivalent phosphate buffer)	Calculated buffering capacity from model polypeptide solutions	Sum of buffering capacities per Fig. 1 (polypeptides and equivalent phosphate buffer)	Experimentally determined total buffering capacity
Target sulfatase	0.6	5.0	5.6	5.1
Second sulfatase	0.6	7.2	7.8	8.1
BSA		8.2	8.2	6.8

It should be noted that ratio of the buffering capacity of the second to target sulfatase calculated with surface-exposed amino acid was 1.6 (experimental value: 2.2) or 1.3 (experimental value: 1.2) during acid or base titration, respectively. While some of the differences could be attributed to experimental variability, the following several factors should also be considered that may partially explain the discrepancies between the calculated and experimental values: first, the pKa of the Asp, Glu, and His residues could vary depending on their extent of solvent exposure, local chemical environment, and lack of any meaningful tertiary structure^16,19,20^; second, the number of surface exposed amino acid derived from crystal structure may not be actually solvent accessible; third, although negligible at individual level, the side chains of other amino acid residues such as arginine, lysine, tyrosine, and cysteine, may collectively contribute to the overall buffering capacity; and lastly, the polypeptides used in this study were polydisperse in nature, introducing further error when calculating their molar concentrations, and, therefore, estimating the final numbers of terminal amine and carboxyl groups. Nonetheless, the simplified calculations of buffering capacity using the number of solvent exposed amino acid residues and buffering capacity of polypeptide solutions provided supportive data at least in the case of our study. Another factor to consider is that the second sulfatase used in the study was treated at lab scale while the actual residual phosphate ICP-MS data were generated using the protein that were manufactured at scale.

In addition to the buffering capacity studies of the non-stressed target sulfatase, the studies were repeated for the stressed target sulfatase. Interestingly, the stressed sulfatase demonstrated an almost identical buffering capacity to its native counterpart despite aggregation and activity loss (data not shown). The secondary structure of the stressed sulfatase was examined by far UV CD, and the tertiary structure was examined by near UV CD. The data suggested some type of fingerprint tertiary conformational change (Figure S2). Nonetheless, the observed conformational changes were not significant enough to affect (1) the overall buffering capacity or more than likely the surface-exposed amino acids composition, i.e. Asp, Glu, and His composition, and (2) the residual buffering capacity attributed to the residual phosphates.

One great advantage of self-buffered formulation is the elimination of additional buffering excipients, such as histidine, citrate, and acetate. These excipients introduce significant challenges during formulation and process development as summarized by Garidel et al. and Karow et al.^7,8^ and therefore may not be appropriate for highly specialized delivery routes such as direct introduction into the CNS system^1,2^. Therefore, the elimination of buffering excipient in this study is particularly advantageous for the sulfatase formulation intended for direct injection into the CNS. Any unnecessary excipient should be avoided to minimize disruption of the delicate balance of the cerebrospinal fluid in the CNS. The findings from current study demonstrated the feasibility of self-buffered formulations and may be explored as an alternative option to the conventional formulation approaches, when appropriate.

## Conclusions

The data presented in this study demonstrated that the self-buffering capacity is not restricted to mAbs; it was also observed for enzymes (sulfatase family) and BSA. The two sulfatases explored in this study belong to the same family; it is highly possible that the conformational structure of the molecules (dimer to multimer equilibrium), presence and potential coordination of a divalent metal ion to residual phosphates as well as the potential residual phosphates on the molecular structure may all have played a role in the observed strong self-buffering capacity in the case of the two sulfatases. Most importantly, the results of the stability studies demonstrated that the sulfatase remained stable for up to 48 months at the long-term storage condition with no significant change to any critical quality attribute. There was no change in the potency of the sulfatase and the structural integrity of the molecule was maintained (as determined by SDS-PAGE, peptide map, glycan map, and HPLC assays) following long-term storage at this temperature. Additionally, the results of the freeze–thaw stability studies demonstrate that the sulfatase is not susceptible to freeze–thaw induced degradation when frozen and thawed at a controlled rate or when thawed at either ambient temperature conditions, 5 ± 3 °C, or water bath set at 37 °C in the absence of any buffering species. The self-buffering capacity was also maintained during in-use and agitation studies. It is noteworthy that throughout the sulfatase’s manufacturing process and history of development, the pH values for the two sulfatases have remained stable. Cumulatively, the data provide confidence regarding the buffering ability of the high concentration sulfatases that are specifically targeted for delivery into the IT space.

## Supplementary Information

Supplementary Information.

## Data Availability

Data generated or analyzed during this study and not included in this manuscript (and its Supplementary Information files) can be shared with the Editorial Board Members and referees on reasonable request to the corresponding author.
