# Supplementary material for: Self-buffering capacity of a human sulfatase for central nervous system delivery
Source: Sci Rep. 2021 Mar 24;11:6727. doi: 10.1038/s41598-021-86178-2 (PMC7991414; doi:10.1038/s41598-021-86178-2)
Supplement: Supplementary file 1 — Supplementary Information. [file 41598_2021_86178_MOESM1_ESM.docx]

**Self-Buffering Capacity of a Human Sulfatase for Central Nervous System Delivery**

Yi Wen^*,#^, Nazila Salamat-Miller^*,†^, Keethkumar Jain, and Katherine Taylor

Shire Pharmaceuticals (a subsidiary of Takeda Pharmaceutical Company), 200 Shire Way, Lexington MA 02421

*Equal contribution as the first authors

^#^Current address: Lilly Research Laboratories, Eli Lilly and Company, Indianapolis IN 46285

^†^Corresponding author: [nazila.miller@takeda.com](mailto:nazila.miller@takeda.com)

**Table S1 - Equivalent Phosphate Buffer for the Target Sulfatase at Different Protein Concentrations**

| Target sulfatase | | Equivalent phosphate buffer |
| --- | --- | --- |
| (mg/mL) | mM | mM |
| 40 | 0.7 | 1.05 |
| 30 | 0.525 | 0.79 |
| 20 | 0.35 | 0.52 |
| 10 | 0.175 | 0.26 |
| 5 | 0.0875 | 0.13 |

**Table S2 - Concentrations of Phosphorus and Cation in ppm Determined by ICP-MS**

| Lot No. | Phosphorus (ppm) | Cation (ppm) |
| --- | --- | --- |
| Lot 1 | 27 | 11 |
| Lot 2 | 31 | 14 |
| Lot 3 | 31 | 15 |
| Lot 4 | 27 | 12 |
| Mean±SD | 29±2.3 | 13±1.8 |

**Table S3 - Analysis of Buffering Capacity with Regards to Total and Surface Exposed Amino Acid residues (Asp, Glu, and His)**

The two sulfatases at 0.5 mM were used as an example for the presented comparison

|  | **0.5 mM** | **Experimentally- Determined Total Buffering Capacity** | | **Buffering Capacity of the Associated Phosphates (Estimated from Equivalent Phosphate Buffer)** | | **Buffering Capacity Contributed by Amino Acids**  **(Total-Phosphate)** | | **Number of Amino Acids** | | **Number of Surface Exposed Amino Acids** | | **Calculated Buffering Capacity from Polypeptides** | | | |
| --- | --- | --- | --- | --- | --- | --- | --- | --- | --- | --- | --- | --- | --- | --- | --- |
|  |  |  |  |  |  |  |  |  |  |  |  | **All Amino Acids** | | **Surface Exposed Amino Acids** | |
| **Protein** | mg/mL | Acid | Base | Acid | Base | Acid | Base | Asp+Glu+His | His | Asp+Glu+His | His | Acid | Base | Acid | Base |
| **Target sulfatase** | 28.3 | 2.4 | 2.7 | 0.14 | 0.42 | 2.2 | 2.3 | 63 | 18 | 36 | 10 | 6.7 | 2.0 | 3.9 | 1.1 |
| **Second sulfatase** | 39.4 | 5.0 | 3.1 | 0.14 | 0.42 | 4.9 | 2.7 | 79 | 15 | 59 | 13 | 7.3 | 1.7 | 5.7 | 1.5 |
| **BSA** | 33.3 | 4.1 | 2.8 | NA. | NA | 4.1 | 2.8 | 115 | 17 | 87 | 8 | 10.4 | 1.9 | 7.3 | 0.9 |
| **Second sulfates to target sulfates ratio** |  | 2.1 | 1.1 |  |  | 2.2 | 1.2 | 1.3 | 0.8 | 1.6 | 1.3 | 1.1 | 0.8 | 1.5 | 1.3 |

**Figure S1 - Titration Curves of Equivalent Phosphate Buffering Solutions at Different Concentrations When Titrated with HCl (Panel a) or NaOH (Panel b)**

**Figure S2 - Near UV and Far UV CD Spectra of Native and Stressed Sulfatase**

**Figure S3 - Titration Curves for Polypeptides without Buffering Capacity at Different Concentrations When Titrated with HCl or NaOH**

**Figure S4 - Titration Curves of the Second Sulfatase Solutions at Different Concentrations When Titrated with HCl or NaOH**

**Figure S5 - Titration Curves of BSA Solutions at Different Concentrations When Titrated with HCl or NaOH**
